# The usefulness of magnetic resonance imaging of the hand and wrist in very early rheumatoid arthritis

**DOI:** 10.1186/ar3355

**Published:** 2011-06-09

**Authors:** Paraskevi E Kosta, Paraskevi V Voulgari, Anastasia K Zikou, Alexandros A Drosos, Maria I Argyropoulou

**Affiliations:** 1 Department of Clinical Imaging and Radiology Medical School, University of Ioannina, 45110, Ioannina, Greece; 2 Rheumatology Clinic, Department of Internal Medicine, Medical School, University of Ioannina, 45110, Ioannina, Greece

## Abstract

**Introduction:**

Magnetic resonance imaging (MRI) was used to study the hand and wrist in very early rheumatoid arthritis (RA), and the results were compared with early and established disease.

**Methods:**

Fifty-seven patients fulfilling the new American College of Rheumatology criteria for RA, 26 with very early RA (VERA), 18 with early RA (ERA), and 13 with established RA (ESTRA), (disease duration < 3 months, < 12 months, and > 12 months, respectively) were enrolled in the study. MRI of the dominant hand and wrist was performed by using fat-suppressed T2-weighted and plain and contrast-enhanced T1-weighted sequences. Evaluation of bone marrow edema, synovitis, and bone erosions was performed with the OMERACT RA MRI scoring system.

**Results:**

Edema, erosions, and synovitis were present in VERA, and the prevalence was 100%, 96.15%, and 92.3%, respectively. Significant differences in edema and erosions were found between VERA and ESTRA (*P *< 0.05). No significant difference was found in synovitis.

**Conclusions:**

Edema, erosions, and synovitis are findings of very early RA. MRI, by detecting these lesions, may play an important role in the management of these patients.

## Introduction

Rheumatoid arthritis (RA) is a chronic systemic inflammatory disease characterized by prominent joint manifestations. Inflammation of the synovial membrane leads to the formation of a highly cellular inflammatory tissue, the pannus, which, by eroding cartilage and bone, leads to joint destruction and ankylosis [1]. Articular involvement of the hand and wrist has been considered a very frequent presenting finding [1]. The presence of marginal erosions, seen on conventional radiographs of the hand and wrist, has been viewed as a specific and relatively sensitive diagnostic finding [2]. Conventional radiographs cannot assess synovitis, bone edema, and early marginal erosions [3-8]. Bone edema, erosions, and synovitis have been detected by magnetic resonance imaging (MRI) in patients with disease duration of < 1 year [3,4,8-15]. Bone edema and erosions are considered red flags for progression of bone damage in the future, and thus, modern concepts in RA imply that treatment with conventional disease-modifying antirheumatic drugs (DMARDs) and particularly biologic DMARDs, should ideally be started before erosive disease is detected [16-20]. Nevertheless, no studies have evaluated with MRI the hand and wrist of patients with disease duration of less than 3 months.

The purpose of this study was to assess with MRI in very early RA (VERA) the prevalence and severity of hand and wrist involvement and to compare the involvement with early RA (ERA) and established RA (ESTRA).

## Materials and methods

### Patients

**Figure 1:**
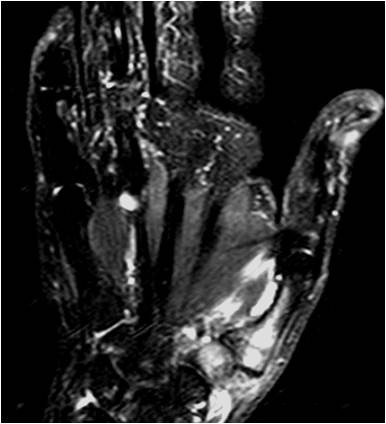
**Coronal short time inversion recovery scan shows edema in trapezium and the first metacarpal bone**. Repetition time/2,500 ms, echo time/60 ms, inversion time/160 ms.

**Figure 2:**
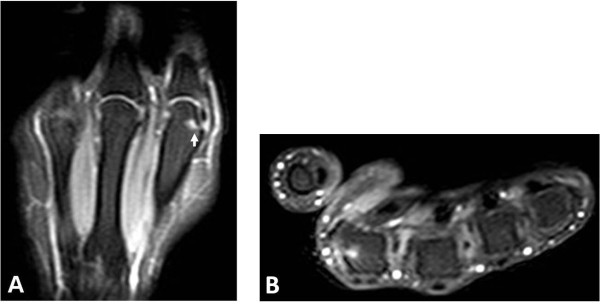
**Fat-suppressed T1-weighted scans (A) coronal and (B) axial plane show an enhanced bone erosion (arrow)**. Rrepetition time/500 msec, echo time/16 msec. Enhanced bone erosion (arrow) in the head of the second metacarpal.

**Figure 3:**
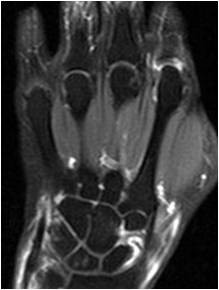
**Coronal T1-weighted fat-suppressed scan shows enhancement of the inflammatory synovium in hand and wrist joints**. Repetition time (TR)/500 msec, echo time (TE)/16 msec.

Fifty-seven consecutive unselected patients with RA, according to the new criteria for RA [2] and without prior use of DMARDs, were enrolled in the study. The 37 women and 20 men were aged 17 to 83 years (mean, 57.52 ± 15.82 years). According to disease duration, the patients were divided into three groups: Group 1, 26 patients with very early disease (< 3 months) (VERA); Group 2, 18 patients with early disease (< 12 months) (ERA); and Group 3, 13 patients with established disease (> 12 months) (ESTRA). Each patient underwent a complete physical examination by the same rheumatologist (PVV). Clinical disease variables included the duration of morning stiffness (minutes), grip strength (mm Hg), total joint count with tenderness or swelling, number of swollen joints, number of tender joints, and pain score (on visual analogue scale (VAS; centimeters)). Laboratory disease variables included C-reactive protein (CRP), erythrocyte sedimentation rate (ESR), rheumatoid factor (RF), and anticitrullinated cyclic peptide (CCP). For assessing disease activity, the disease activity score for 28 joint indices (DAS-28) was calculated [21]. An MRI of the dominant hand and wrist was performed in the same MRI unit (1.5 Tesla; Gyroscan ACS NT; Philips Medical Systems, Best, The Netherlands) by using a surface coil with a 20-cm field of view. The patient lay prone with the arm to be studied extended overhead toward the midline. The imaging protocol consisted of axial and coronal STIR images with 2,500, 60, 160 (repetition time, ms/echo time, ms/inversion time, ms) 3-mm slice thickness, 0.3-mm intersection gap, 256 × 256 imaging matrix, coronal spin-echo T1-weighted images with 500, 16 (repetition time, ms; echo time, ms) 3-mm slice thickness, 0.3-mm intersection gap, 256 × 256 imaging matrix; and coronal spin-echo fat-suppressed, T1-weighted images with 500, 16 (repetition time, ms/echo time, ms) 3-mm slice thickness, 0.3-mm intersection gap, 256 × 256 imaging matrix before and coronal and axial images immediately after intravenous administration of 0.1 mmol/kg Gd-DTPA. Intravenous contrast injection was performed through a vein in the contralateral arm. Diffusion of contrast material into joint effusions was avoided in coronal scans performed immediately after contrast administration (duration of postcontrast coronal sequence, 2.5 minutes). Hand and wrist involvement was evaluated by using the OMERACT RA MRI scoring system to assess bone edema (Figure 1), erosions (Figure 2); and synovitis (Figure 3) [22,23]. Evaluations of all MRI examinations were performed independently by two musculoskeletal radiologists (PEK, AKZ) blinded to the patients' identity, clinical status, and disease duration. STIR images were evaluated for bone edema, and plain and contrast-enhanced fat-suppressed T1-weighted coronal and axial images were evaluated for erosions and synovitis. Because the thinnest slice that could be obtained was 3 mm, attention was paid to avoid considering as bone erosions areas of irregular bone contours or ligamentous attachments [24]. The study was approved by the Institutional Review Board, and written informed consent was obtained from all subjects.

### Statistical analysis

Statistical analysis was performed with SPSS base 15 for Windows. Interobserver variability was evaluated by using the Pearson correlation coefficient. Analysis of variance (ANOVA), followed by the least significant difference test, was used to study differences between groups. A *P *value less than 0.05 was considered statistically significant.

## Results

The clinical features of the patients are shown in Table 1. The interobserver variability was R = 0.95 for edema, 0.95 for erosions, and 0.97 for synovitis. Bone edema, erosions, and synovitis were present in all groups of RA (Table 2). Analysis of variance demonstrated that the three groups were significantly different for bone edema and erosions. The least significant difference test revealed significant difference in bone edema and erosions between VERA and ESTRA; *P *< 0.05 (Table 3). Comparison between VERA and ERA in terms of local distribution of lesions revealed significant difference in erosions of the MCP (Table 4).

**Table 1:** Clinical data in 57 patients with very early, early and established rheumatoid arthritis

	VERA (*n *= 26)	ERA (*n *= 18)	ESTRA (*n *= 13)
Age, mean (SD), yr	56.15 (16.58)	61.86 (15.61)	60.40 (10.57)
Female/male	16/10	13/5	8/5
Disease duration, mean (SD), mo	1.65 (0.89)	7.83 (3.29)	33.30 (4.28)
Disease activity score for 28 joint indices, mean (SD)	5.55 (1.20)	5.25 (0.86)	5.50 (1.00)
C-reactive protein, mean (SD)	24.54 (10.50)	17.66 (11.62)	20.71 (10.80)
Rheumatoid factor positivity	19/26	12/18	9/13
Anticitrullinated cyclic peptide positivity	18/26	12/18	9/13

Demographic, clinical, and laboratory data in 57 patients with very early rheumatoid arthritis (VERA; disease duration, < 3 months), early rheumatoid arthritis (ERA; disease duration, < 12 months), and established rheumatoid arthritis (ESTRA; disease duration, > 12 months)

**Table 2:** Prevalence of edema, erosions, and synovitis (evaluated with the OMERACT RA MRI scoring system)

	VERA (%)	ERA (%)	ESTRA (%)
Edema	100	93.33	100
Erosions	96.15	93.33	100
Synovitis	92.3	80	100

Prevalence of bone edema, erosions, and synovitis (evaluated with the OMERACT RA MRI scoring system) in the three groups of patients with very early rheumatoid arthritis (VERA; disease duration, < 3 months), early rheumatoid arthritis (ERA; disease duration, < 12 months), and established rheumatoid arthritis (ESTRA; disease duration, > 12 months)

**Table 3:** Analysis of variance with total score for bone edema, erosions and synovitis

	VERA	ERA	ESTRA	*F*	*P *value
Bone edema	15.5 (11.8)	16.5 (9.1)	24.4 (12.8)	2.31	< 0.05
Bone erosions	6.0 (5.2)	7.3 (5.09)	10.5 (7.02)	2.36	< 0.05
Synovitis	10.1 (5.2)	11.2 (4.5)	12.5 (3.3)		NS

Values represent the mean (± SD). Analysis of variance between the three groups, with total score for bone edema and erosions depicted with magnetic resonance imaging of the hand in three groups of patients with very early rheumatoid arthritis (VERA; disease duration, < 3 months), early rheumatoid arthritis (ERA; disease duration, < 12 months), and established rheumatoid arthritis (ESTRA; disease duration, > 12 months)

Analysis of variance demonstrated that the three groups were significantly different. Least significant difference test revealed significant difference in bone edema and erosions between VERA and ESTRA.

**Table 4:** Distribution of edema, erosions and synovitis in hand and wrist joints

	MCP		CMC		WRIST		RADIOULNA	
	**VERA**	**ERA**	**VERA**	**ERA**	**VERA**	**ERA**	**VERA**	**ERA**

Edema	5.0 (5.9)	4.0 (3.8)	2.7 (2.2)	2.7 (2.5)	7.1 (4.5)	8.3 (3.4)	0.6 (1.2)	1.3 (1.5)
Erosions	0.8(1.3)^a^	1.9(1.7)^a^	1.3(1.6)	1.2 (1.7)	3.4 (3.0)	4.4 (4.2)	0.5 (0.8)	0.9 (1.6)
Synovitis	5.3 (3.4)	4.4 (3.5)			4.6 (2.6)	4.9 (3.4)		

Distribution of bone edema, erosions, and synovitis in metacarpophalangeal joints (MCP), base of metacarpal (CMC), wrist joints, and radioulnar joint area in patients with very early rheumatoid arthritis (VERA; disease duration, < 3 months), and early rheumatoid arthritis (ERA; disease duration, < 12 months).

The distribution of erosions in separate joint areas in patients with VERA and ERA revealed significant difference only in MCP joints (^a^*P *< 0.05). No difference was found in bone edema and synovitis.

Data are presented as mean (SD).

No significant correlation was found between the imaging findings and the clinical (duration of morning stiffness (min), grip strength (mm Hg), total joint count with tenderness or swelling, number of swollen joints, number of tender joints and pain score on VAS (cm)) and laboratory findings (CRP, ESR, RF, anti-CCP, and DAS-28).

## Discussion

In this study, the OMERACT RA MRI scoring system was applied to look for differences in bone edema, erosions, and synovitis between VERA, ERA, and ESTRA, and the major findings were (a) the presence of bone edema, erosions, and synovitis at the very early stages of RA; and (b) a significant difference in bone edema and erosions between VERA and ESTRA.

MRI is being used largely in the assessment of hand and wrist involvement of patients with RA [3,5-7,13-16]. Most of the studies have been performed in patients with late early and established disease. Only one study evaluated patients with disease duration less than 4 months [4]. This study demonstrated an incidence of bone erosions of 45%, but half of the patients were receiving DMARDs. The present study demonstrated a very high incidence of bone erosions (96%) in a treatment-naïve population evaluated at a very early stage of RA. The lack of treatment and the evaluation of the MCP joints, which are characteristically affected in early RA [14], may probably explain the difference in incidence of bone erosions. Previous studies, by demonstrating very early the presence of anti-CCP antibodies and RF, have suggested that the disease process in RA starts long before the onset of symptoms [25]. This study, by demonstrating bone erosions in VERA, reinforces this hypothesis. Another interesting finding of this study was the presence of bone edema in all patients with VERA. Bone edema is a pre-erosive lesion that represents true inflammation and can be seen on MRI alone or surrounding bone erosions [24]. Bone edema can be present at any stage of RA and has been associated with more-aggressive disease [26,27]. Lack of treatment that could decrease the aggressiveness of the disease process might probably explain the presence of bone edema in all patients with VERA.

In this study, the presence of synovitis was independent of the disease duration, and the incidence was high in all groups. In the VERA group, the incidence of synovitis and bone erosions was almost similar. The exact nature of the relation between synovitis and bone damage remains unclear. The synovium seems to be the prime target in the inflammatory course of RA. Conaghan *et al *[12] showed that in ERA, synovitis appears to be the primary abnormality, and bone damage occurs as a late effect in proportion to the level of synovitis but not in the absence of synovitis. Bone erosions are caused by direct invasion of pannus into bone but also by pro-osteoclastogenic imbalance, which is cytokine driven. The pathogenic mechanism in the very early period of RA may be different from that in established disease. Current theories of the immunopathogenesis of RA suggest that abnormally sensitive to tumor necrosis factor (TNF)-α bone marrow stem cells could travel via the systematic circulation to the subchondral bone marrow, where they initiate inflammatory and pre-erosive changes or could travel to the synovial membrane, where they promote synovial hyperplasia and inflammatory synovitis. It seems that bone marrow edema represents a true inflammation in the bone and is a pre-erosive lesion that can be reversible. Irreversibility of bone edema with increasing disease chronicity is probably due to organization of the inflammation with formation of dense vascularized infiltration and activation of osteoclasts. Thus, in contrast to radiographic erosions that reflect bone damage that has already occurred, bone marrow edema represents early inflammatory infiltrates in the subchondral bone [27-29].

In the current study, a significant difference in edema and erosions was demonstrated between VERA and ESTRA. This is in agreement with previous studies, which, by using hand radiographs, showed that up to 60% of the patients develop joint erosions at the end of the 1 year from disease onset [30]. The present study did not reveal any relation between the MRI findings in ERA and disease activity. A recent study performed in a 3-T MR unit by using a dynamic contrast-enhanced T1-weighted sequence demonstrated a correlation between synovitis and DAS-28. This study was performed in a small series of patients, and only synovitis was evaluated [31]. Further studies are needed in larger series of patients to assess the usefulness of 3-T MRI in the detection of hand-wrist lesions in patients with ERA.

## Conclusions

In conclusion, bone edema, erosions and synovitis are very early MRI findings of RA. MRI of the hand and wrist on clinical diagnosis of RA is useful to assess the degree of involvement.

## Abbreviations

ANA: antinuclear antibodies; ANOVA: analysis of variance; CCP: citrullinated cyclic peptide; CRP: C-reactive protein; DAS-28: disease activity score of 28 joint indices; DMARDs: disease-modifying antirheumatic drugs; ERA: early rheumatoid arthritis; ESR: erythrocyte sedimentation rate; ESTRA: established rheumatoid arthritis; MCP: metacarpophalangeal; MRI: magnetic resonance imaging; RA: rheumatoid arthritis; RF: rheumatoid factor; TNF: tumor necrosis factor; VAS: visual analogue scale; VERA: very early rheumatoid arthritis.

## Competing interests

The authors declare that they have no competing interests.

## Authors' contributions

PEK helped to establish imaging techniques, analyze data, interpret data, and draft the manuscript. AKZ perform the examinations and analyzed data. MIA reviewed the interpreted data and critically reviewed the manuscript. PVV selected the patients and performed the musculoskeletal examination. AAD designed the study and critically reviewed the manuscript. All authors read and approved the final version of the manuscript.
